# Takotsubo cardiomyopathy in endurance athletes: Clinical insights from marathon-related cases

**DOI:** 10.21542/gcsp.2026.6

**Published:** 2026-02-28

**Authors:** Husam Katib, Ngoc Thai Kieu, Sabeeh Islam

**Affiliations:** Jacobs School of Medicine & Biomedical Science, University at Buffalo, Buffalo, NY 14260, USA

## Abstract

**Background:** Takotsubo cardiomyopathy (TTC), or stress-induced cardiomyopathy, is characterized by transient left ventricular dysfunction often triggered by emotional or physical stress. Although classically associated with postmenopausal women under psychological stress, cases among endurance athletes, particularly marathon runners, have emerged, suggesting a unique pathophysiological link between extreme exercise, catecholamine surge, and microvascular dysfunction.

**Objective:** This review synthesizes published cases and mechanistic studies of TTC occurring in endurance athletes, emphasizing marathon-related presentations, diagnostic challenges, and clinical implications.

**Methods:** A structured literature review of PubMed, Scopus, and Web of Science identified case reports and series meeting predefined diagnostic and methodological criteria. Dual extraction, quality appraisal, and PRISMA-guided selection were performed.

**Results:** A total of 42 documented cases were analyzed. The majority occurred in middle-aged athletes, predominantly female, with acute chest pain or dyspnea immediately after race completion. Troponin elevations were modest, and coronary angiography was negative for obstructive lesions. Echocardiography and cardiac MRI typically demonstrated apical or mid-ventricular ballooning with rapid recovery. Mechanistic data support transient catecholamine-mediated myocardial stunning and microvascular dysfunction as central features.

**Conclusion:** TTC in endurance athletes represents a distinct clinical entity within the spectrum of stress cardiomyopathies. Recognition of this condition is crucial in differentiating it from acute coronary syndromes and preventing recurrence through risk modification and psychological or autonomic management.

## Introduction

Takotsubo cardiomyopathy (TTC), first described in Japan in 1990, is characterized by transient regional left ventricular systolic dysfunction that often mimics acute myocardial infarction but occurs in the absence of obstructive coronary artery disease^1^. Although initially considered rare, the syndrome now accounts for approximately 1–2% of all presentations initially diagnosed as acute coronary syndrome^2^. Classically, TTC affects postmenopausal women after severe emotional stress, such as bereavement or anxiety; however, an increasing number of reports describe TTC following intense physical exertion, including marathon running, triathlon participation, and other endurance events^3–5^.

Endurance exercise induces a massive catecholaminergic response, oxidative stress, and transient coronary microvascular constriction^6^. In susceptible individuals, this physiological surge may lead to myocardial stunning, reversible microvascular ischemia, and ultimately TTC. Recognition of TTC in athletes is challenging because the clinical picture often overlaps with ischemic syndromes, and many patients are otherwise young and healthy^7^. Understanding the unique triggers, pathophysiology, and prognosis in this population can aid early recognition and management while preventing unnecessary interventions. This review integrates available evidence and case data regarding TTC in endurance athletes, focusing on marathon-related events as a model of extreme physiological stress.

## Methods

A structured literature search was conducted across PubMed, Scopus, and Web of Science through September 2024 to identify reports describing Takotsubo cardiomyopathy temporally associated with endurance exercise. The search incorporated combinations of the terms “Takotsubo”, “stress cardiomyopathy”, “marathon”, “endurance exercise”, “ultramarathon”, “triathlon”, “athlete”, “apical ballooning”, and related variations. Reference lists of all eligible studies and review articles were also screened to ensure comprehensive capture of relevant cases. Studies were considered eligible if they described TTC occurring within 24 h of participation in an endurance event lasting at least two hours, such as a marathon, ultramarathon, or triathlon, and if the diagnosis met modified Mayo or InterTAK criteria. Specifically, included cases required documentation of transient left ventricular systolic dysfunction, the absence of obstructive coronary disease, evidence of myocardial stress in the form of elevated cardiac biomarkers or ECG abnormalities, and demonstration of recovery on follow-up imaging. Reports were excluded if symptom onset occurred more than 24 h after exertion, if diagnostic evaluation was incomplete or insufficient to differentiate TTC from myocarditis or ischemic injury, or if an alternative diagnosis was clearly established.

All titles and abstracts identified during the search were screened, followed by full-text review of potentially relevant publications. The selection process followed the PRISMA framework, beginning with 312 records, of which 238 remained after removal of duplicates. Following title and abstract screening, 64 articles underwent full-text evaluation, and 42 met all inclusion criteria. Data extraction was performed independently by two reviewers using a standardized collection form that captured demographic characteristics, training history, comorbidities, event type and conditions, biomarker levels, echocardiographic features, cardiac magnetic resonance findings when available, angiographic results, management strategies, and clinical outcomes. Discrepancies in extraction were resolved through consensus. The methodological quality of included cases was assessed using the Joanna Briggs Institute checklist for case reports, which demonstrated that most articles fulfilled the majority of criteria, though deficiencies related to incomplete reporting of environmental conditions and follow-up intervals were common. Given the heterogeneity in reporting styles, biomarker units, imaging protocols, and follow-up timing, no meta-analysis was attempted, and findings were synthesized descriptively. Potential publication bias was acknowledged, as case reports are more likely to emphasize unusual, severe, or particularly illustrative presentations, and rarely capture the full denominator of exposed individuals.

## Results

Across the 42 published cases included in this review, the median age of affected athletes was 48 years, with a range of 32 to 63 years, and women represented approximately two-thirds of the cohort (64%). Most cases were associated with marathon events, which accounted for 32 of the 42 cases (76%), while ultramarathons contributed 6 cases (14%) and triathlons 4 cases (10%). When reported, athletes typically had several years of structured endurance training prior to their event, with a median training history of 6 years (range 2–19 years). Comorbidities were relatively uncommon but were present in roughly one-fifth of the cohort; these included hypertension, hypothyroidism, and anxiety disorders (Table 1). Environmental stressors were inconsistently documented, yet among the 18 cases that reported such data, ambient temperatures ranged from 22 °C to 31 °C with humidity between 55% and 88%, suggesting that heat and hydration stress may have contributed to physiological strain.

**Table 1:** Clinical and demographic characteristics of TTC in endurance athletes.

Variable	Mean/%/Median	Range/Details
Age (years)	48	32–63
Female sex	64%	—
Event type	Marathon 76%, Ultramarathon 14%, Triathlon 10%	—
Training history	Median 6 years	2–19 years
Comorbidities	22%	HTN 10%, Hypothyroidism 5%, Anxiety 7%
Symptom onset post-event	<1 h	0–60 min
Troponin (ng/mL)	Median 1.08	0.12–7.4
Baseline LVEF	Mean 39%	28–55%
Recovery LVEF	≥50% in 93%	55–68%
Recovery time	Median 6 weeks	2–8 weeks
Method of diagnosis	Echo 100%, CMR 69%, Angiography 100%	—

Troponin concentrations were elevated in all cases but were generally modest compared to typical acute myocardial infarction. The recorded values ranged from 0.12 to 7.4 ng/mL, with a median of 1.08 ng/mL. Baseline left ventricular ejection fraction (LVEF) at presentation demonstrated significant systolic impairment, varying between 28% and 55%, with a mean value of 39%. Recovery of ventricular function was common and occurred relatively quickly: by six to eight weeks, 93% of athletes had achieved LVEF normalization, defined as an ejection fraction of at least 50%. The 7% of athletes whose LVEF remained below 50% at two months typically exhibited apical ballooning variants and had returned to intense training during the early recovery phase, raising concern for premature exertion as a potential contributor to delayed normalization.

Electrocardiographic abnormalities were frequent, occurring in 88% of cases. ST-segment elevation was observed in roughly one-third (38%), whereas T-wave inversion appeared in nearly two-thirds (62%), and QTc prolongation in approximately one-fifth (19%). Echocardiography revealed the classic apical ballooning pattern in the majority (71%), with mid-ventricular variants in 19% and basal (reverse) patterns in 10%. Cardiac magnetic resonance imaging, performed in 29 cases, consistently demonstrated myocardial edema in the acute phase. Late gadolinium enhancement (LGE) was absent in 93% of studies, consistent with the reversible nature of TTC, while transient, mild, patchy LGE occurred in a small minority of athletes but resolved on follow-up.

Coronary angiography was performed in all cases and uniformly demonstrated the absence of obstructive coronary artery disease (Table 2). Clinical outcomes were favorable across the dataset, with no reported deaths and a median hospital stay of three days. Two athletes experienced recurrence of TTC; in both instances, the recurrence followed an early return to competitive endurance events before full physiologic recovery had been confirmed. These findings collectively demonstrate a consistent clinical pattern in exercise-induced TTC: modest troponin elevation, transient systolic dysfunction, characteristic imaging findings, and near-complete recovery when appropriate convalescence is observed.

**Table 2:** Diagnostic findings.

Modality	Findings	%/Quantitative Metrics
ECG	ST-elevation, T-wave inversion, QT prolongation	ST-elev 38%, TWI 62%, QTc prolongation 19%
Troponin	Elevated	Median 1.08 ng/mL (0.12–7.4)
Echocardiography	LV akinesia patterns	Apical 71%, Mid 19%, Basal 10%
CMR	Edema without infarction	Edema 100%; Absence of LGE 93%
Angiography	Normal coronaries	100%
LVEF Recovery	Normalization ≥ 50%	93% recovery; 7% non-normalization at 2 months

## Discussion

### Pathophysiologic mechanisms

The pathophysiology of TTC in athletes is multifactorial, involving excessive catecholamine release, myocardial stunning, and coronary microvascular dysfunction. During endurance exercise, plasma norepinephrine and epinephrine rise up to 10-fold above baseline, resulting in heightened *β*-adrenergic stimulation^8^. Experimental work by Ueyama et al. demonstrated that high catecholamine concentrations cause focal myocardial necrosis and contractile dysfunction resembling TTC^9^. This surge, compounded by dehydration, electrolyte imbalance, and oxidative stress, predisposes the myocardium to calcium overload and transient dysfunction^10^. Several investigators, including Lyon et al. and Redfors et al., have emphasized microvascular constriction and endothelial dysfunction as key mediators linking catecholamine toxicity to regional wall motion abnormalities^11,12^.

### Role of coronary microvascular dysfunction

Recent coronary flow reserve studies and invasive pressure-wire analyses confirm that microvascular dysfunction persists during the acute phase of TTC and recovers parallel to ventricular function^13^. Ekenbäck et al. demonstrated reduced coronary flow reserve and abnormal index of microvascular resistance in TTC compared to controls^14^. Endurance athletes experience repetitive episodes of shear stress and oxidative injury that may impair microvascular vasodilatory capacity over time^15^. Consequently, marathon-related TTC may represent an acute decompensation on a background of cumulative microvascular strain. Schweiger et al. recently highlighted that coronary microvascular dysfunction predicts recurrence and adverse outcomes, underscoring its clinical significance^16^.

### Neuroendocrine and autonomic imbalance

Autonomic imbalance also contributes to TTC pathogenesis. Endurance training modulates vagal tone, yet intense competition often provokes sympathetic dominance, particularly under psychological stress. The interplay between physical exhaustion, competition anxiety, and autonomic instability may explain why TTC occasionally occurs in well-trained athletes rather than novices^17^. Neuroimaging studies reveal transient hypothalamic and amygdalar hyperactivation during TTC episodes, implicating central sympathetic outflow in the cascade^18^.

### Clinical presentation and diagnostic challenges

Clinically, TTC in endurance athletes can closely mimic myocardial infarction. ECG findings, including ST-segment elevation or T-wave inversion, are indistinguishable from ischemic events^19^. Troponin elevation, although mild, often prompts urgent catheterization. Given the low pre-test probability of coronary disease in young athletes, awareness of TTC is essential to avoid unnecessary interventions. Cardiac MRI plays a crucial role by demonstrating edema without scar, confirming reversibility^20^. Advanced echocardiography, including strain imaging, can detect subtle contractile recovery patterns and microvascular involvement^21^.

### Management and prognosis

Treatment is largely supportive, focusing on afterload reduction, *β*-blockade, and careful monitoring. Recovery is typically complete within weeks. However, recurrent TTC has been reported in athletes resuming competition without addressing stress or hydration factors^22^. Beta-blockers may attenuate catecholamine surges during future events. Counseling regarding psychological preparation and moderation of exertional stress may reduce recurrence risk^23^. Prognosis remains favorable, with near-complete functional recovery and very low mortality reported in this subset^24^.

### Integration with broader Takotsubo research

The athletic form of TTC broadens understanding of the syndrome as a spectrum disorder precipitated by both emotional and physical stressors. Compared with classical postmenopausal TTC, athlete-related cases feature higher catecholamine exposure, fewer comorbidities, and faster recovery^25^. Recent registries, including the InterTAK database, suggest that up to 10% of TTC cases involve physical triggers such as exercise^26^. Dong et al. experimentally demonstrated that TTC represents a microvascular disease, supporting the shared mechanism between emotional and physical variants^27^. These insights reinforce the importance of integrating microvascular assessment into future athlete screening and stress testing protocols^28^.

## Clinical Utility

### Pre-event risk stratification, return-to-competition, and race-day risk mitigation

Although TTC in endurance athletes is rare, its overlap with other race-related cardiac events, especially exertional arrhythmias, demand ischemia leading to non-obstructive myocardial infarction, and viral or immune-triggered myocarditis, highlights the need for structured risk assessment and prevention strategies. In marathon populations, sudden cardiac arrest is far more commonly attributable to hypertrophic cardiomyopathy, occult coronary artery disease, ventricular arrhythmias, or myocarditis than TTC, which remains a small fraction of cardiac events documented during races^2,26^. However, TTC-related presentations may be under-recognized due to the transient nature of dysfunction and post-race delays in imaging.

### Pre-event risk stratification for endurance athletes with possible TTC susceptibility

Pre-event evaluation remains central because many athletes who develop TTC exhibit subtle predisposing features, including heightened adrenergic reactivity, recent emotional stress, or cumulative microvascular strain. A targeted approach may include:

• **Baseline echocardiography**, particularly for middle-aged athletes or those with prior unexplained exertional chest symptoms, to detect subclinical diastolic dysfunction or wall-motion variability that may increase vulnerability^19–21^.
• **Coronary microvascular assessment** (e.g., stress perfusion MRI or PET) in athletes with a prior TTC event or exertional chest pain, given the demonstrated prognostic role of microvascular dysfunction^13–16^.
• **Biomarker screening** in the month preceding major events—such as high-sensitivity troponin, BNP/NT-proBNP, and markers of autonomic imbalance—may help identify individuals with ongoing physiological strain, although evidence remains limited.
• **Psychological stress screening**, particularly for athletes undergoing major life stressors, since central autonomic activation plays a key role in TTC pathogenesis^17,18^.

Because TTC may overlap symptomatically with ischemia, athletes with new chest symptoms during training should undergo prompt cardiac evaluation rather than attributing symptoms to “overtraining”.

### Return-to-competition (RTC) protocol after TTC

Return-to-competition decisions should follow structured steps similar to myocarditis but with recognition of the typically benign course of TTC. Based on available case series and expert consensus from TTC management guidelines^23,24^, a practical approach includes:

#### Rest and recovery (0–4 weeks)

• Athletes should avoid high-intensity training until left ventricular ejection fraction normalizes and wall-motion abnormalities resolve.
• *β*-blockers may be continued during early recovery to blunt catecholamine surges^22,23^.

#### Post-recovery evaluation (4–8 weeks)

• Repeat echocardiography with strain imaging to document full functional recovery.
• Consider stress testing with perfusion imaging if symptoms preceded the TTC event or if microvascular dysfunction was documented acutely.

#### Graduated return to training (8–12 weeks)

• Begin low-intensity endurance training and progress in a staged fashion over 4–6 weeks.
• Monitor for recurrence of chest discomfort, dyspnea, or autonomic symptoms such as dizziness or presyncope.

#### Full competitive clearance

• Allowed once LV function is normal, symptoms have resolved, and stress testing shows no inducible ischemia or microvascular abnormalities.
• For athletes with recurrent TTC or persistent autonomic imbalance, long-term *β*-blocker therapy or sports psychology interventions may be appropriate.

This structured pathway stands in contrast to athlete recovery after myocarditis (which typically requires 3–6 months), emphasizing the more favorable reversibility and prognosis of TTC.

### Comparison with other marathon-related cardiac events

From a clinical significance perspective:

• **Incidence**: Myocardial infarction and malignant arrhythmias remain the predominant causes of race-related cardiac arrest. TTC is rare but likely underreported due to transient findings and rapid post-race dispersal.
• **Severity**: Unlike acute MI or acute myocarditis—which carries measurable morbidity—TTC typically exhibits rapid recovery, minimal troponin release, and low risk of long-term LV dysfunction.
• **Pathophysiology**: TTC is uniquely linked to catecholaminergic myocardial stunning and microvascular dysfunction rather than fixed coronary obstruction or inflammatory myocardial injury.
• **Prognosis**: Athlete TTC has a markedly better prognosis and lower recurrence risk compared to traditional emotional-stress TTC, myocardial infarction, or myocarditis.

Placing TTC within this broader context emphasizes that while marathon-triggered TTC is clinically dramatic, it is less dangerous than most alternative diagnoses and responds predictably to supportive care.

### Future directions

Future studies should evaluate the role of pre-race biomarkers, wearable telemetry, and stress imaging to predict vulnerability to TTC in endurance athletes. Prospective multicenter data are needed to quantify incidence and recovery kinetics. Understanding genetic predisposition, particularly *β*-adrenergic receptor polymorphisms, may help identify at-risk individuals^29,30^. Incorporating advanced perfusion imaging and AI-based strain analysis could further refine diagnosis and differentiate TTC from myocarditis or ischemic injury in sports cardiology practice^31^.

### Limitations of current study

Several important limitations must be acknowledged in interpreting the results of this synthesis. Because the dataset is derived entirely from published case reports and small case series, the findings are highly susceptible to publication bias, with a tendency for more dramatic or unusual presentations to be reported, while milder or rapidly resolving cases may go undocumented. The absence of a denominator population also prevents estimation of the true incidence of TTC among endurance athletes, making it impossible to determine whether the observed cases represent a rare complication or an underrecognized phenomenon. Diagnostic heterogeneity further complicates interpretation, as not all reports strictly adhered to InterTAK or modified Mayo criteria, and variability in the timing and modality of imaging may influence the characterization of ventricular dysfunction and recovery.

Moreover, the biomarker and imaging data included in the reports were inconsistent, with different troponin assays, reporting units, and echocardiographic protocols, limiting the ability to compare values across cases quantitatively. Environmental conditions, hydration status, psychological stressors, and pacing strategies (factors likely relevant to TTC pathogenesis) were reported in only a minority of cases, restricting conclusions regarding their role. The causal relationship between endurance exercise and TTC, although strongly suggested by the temporal association in these reports, cannot be definitively established, as unmeasured emotional or physiological stressors may have contributed. Misclassification remains possible, particularly in distinguishing TTC from myocarditis, coronary spasm, or plaque rupture in cases with incomplete ancillary testing. Finally, the geographic and demographic makeup of published cases was relatively narrow, with most reports originating from Europe, North America, and East Asia, raising questions about global generalizability.

## Conclusion

Takotsubo cardiomyopathy in endurance athletes represents a distinctive, reversible form of stress-induced myocardial dysfunction. Marathon-related TTC highlights the complex interaction between catecholamine excess, microvascular dysfunction, and autonomic imbalance. Prompt recognition and appropriate imaging are essential to differentiate it from ischemic events and ensure safe recovery. Preventive strategies including *β*-blockade, hydration, and stress management may mitigate recurrence. Broader awareness among clinicians, sports physicians, and event organizers can improve outcomes and guide evidence-based participation advice for competitive athletes.
